# Chromogranin A cells in the stomachs of patients with sporadic irritable bowel syndrome

**DOI:** 10.3892/mmr.2014.2472

**Published:** 2014-08-08

**Authors:** MAGDY EL-SALHY, ODD HELGE GILJA, TRYGVE HAUSKEN

**Affiliations:** 1 Section for Gastroenterology, Department of Medicine, Stord Helse-Fonna Hospital, Stord 54 09, Norway; 2 Section for Gastroenterology, Department of Clinical Medicine, University of Bergen, Bergen 5020, Norway

**Keywords:** computer image analysis, endocrine cells, immunohistochemistry, immunoreactivity intensity

## Abstract

Several abnormalities have been demonstrated in the intestines of patients with irritable bowel syndrome (IBS); however, the endocrine cells in the stomachs of these patients have not been investigated. The aim of the present study was to determine whether there are any abnormalities in the endocrine cells of the stomachs of patients with IBS using chromogranin A (CgA) as a common marker for endocrine cells. A total of 76 patients were included, of which 26 presented with diarrhoea as the predominant symptom (IBS-D), 21 exhibited diarrhoea and constipation (IBS-M), and 29 experienced constipation as the predominant symptom (IBS-C). In addition, 59 healthy volunteers were recruited as controls. The patients and the controls underwent gastroscopy, and biopsy samples were obtained from the antrum and corpus of the stomach. The biopsy samples were immunostained and the CgA-positive cell density and the intensity of the CgA immunoreactivity were determined. The CgA-positive cell densities in the antra of patients with IBS-M were significantly reduced relative to the controls (P<0.01), while the densities were significantly increased in the antra and corpora of the IBS-C patients (P<0.01 and P<0.001 respectively). The intensities of CgA immunoreactivity did not differ significantly between the IBS patients and the controls. The abnormalities in the densities of endocrine cells were not associated with concomitant changes in the intensities of immunoreactivity; this may indicate unchanged synthesis and/or release of the hormones. In conclusion, the difference in the density of endocrine cells among the IBS subtypes may reflect a role of these cells in the differential symptomologies of these subtypes.

## Introduction

Irritable bowel syndrome (IBS) is a common chronic gastrointestinal disorder with a prevalence of 5–20% and an incidence of ~200 per 100,000 individuals (1). There are currently no radiological, biochemical or endoscopic markers for the diagnosis of IBS (2), which is therefore mainly diagnosed by assessing the clinical symptoms of (3,4): Abdominal discomfort/pain, altered bowel habits and bloating/abdominal distension (5,6).

While IBS does not develop into serious diseases (for example, colon cancer) and is not associated with increased mortality rates (7,8), it considerably reduces quality of life, and is an economic burden to the patient and society due to direct and indirect costs (1). The direct costs are attributable to the evaluation and treatment of the condition, and the indirect costs may be due to absenteeism from school or work, or presenteeism resulting in reduced productivity while at school or work (1).

Chromogranin A (CgA) was first isolated from secretory granules from the bovine adrenal medulla (9,10). CgA is co-stored and co-released with monoamines and peptide hormones from various sites, including the endocrine cells of the gastrointestinal tract (9,10). CgA is commonly used as a marker for gut endocrine cells and endocrine tumours (9–11).

Changes in the population of CgA-immunoreactive endocrine cells, as a marker for the entire population of the endocrine cells, have been investigated in the duodenum, ileum, colon and rectum of IBS patients (12–14). Whereas the density of CgA-immunoreactive cells is reduced in the duodenum, ileum and colon, it does not differ from that of healthy subjects in the rectum. To the best of our knowledge, alterations in the population of CgA-immunoreactive cells in the stomach of patients with IBS have not been investigated. Therefore, the present study was undertaken to identify any changes in the total population of endocrine cells in the stomach, as identified by CgA immunoreactivity.

## Materials and methods

### Patients and controls

A total of 76 patients who fulfilled Rome III Criteria (3,4) for IBS were included in the study. These patients comprised 62 females and 14 males with a mean age of 32 years (range, 18–55 years). The predominant IBS symptoms were diarrhoea (IBS-D), present in 26 patients, diarrhoea and constipation (IBS-M) in 21 patients, and constipation (IBS-C) in 29 patients. All patients had suffered from IBS symptoms for a number of years and none were able to associate the onset of these symptoms with any particular event, such as a gastrointestinal infection. All patients underwent a complete physical examination and were submitted to blood analysis of the following parameters: Full blood count, electrolytes, inflammatory markers, and liver and thyroid function.

Healthy volunteers without any gastrointestinal complaints were recruited as controls via a local announcement at Stord Hospital (Stord, Norway), Haukelands University Hospital (Bergen, Norway) and the University of Bergen (Bergen, Norway), as well as in local newspapers. A total of 59 healthy subjects were included: 15 were from the population of Stord city, and 44 were students or hospital employees. The controls comprised 43 females and 16 males with a mean age of 38 years (range, 20–67 years).

The study was performed in accordance with the Declaration of Helsinki and was approved by the Committee for Medical Research Ethics at the University of Bergen (Bergen, Norway). All subjects gave oral and written consent to participate in the study.

### Gastroscopy, histopathology and immunohistochemistry

Following an overnight fast, the patients and controls underwent standard gastroscopy, during which three biopsy samples were obtained from the antrum and three from the corpus (major curvature) of the stomach. Two additional biopsy samples were removed from the antrum and used for a rapid urease test to identify the presence of *Helicobacter pylori*.

The biopsy samples were fixed overnight in 4% buffered paraformaldehyde, embedded in paraffin and cut into 5-μm sections. The sections were stained with haematoxylin and eosin and immunostained using the avidin-biotin complex (ABC) method with the Vectastain ABC kit (Vector Laboratories, Burlingame, CA, USA). The primary antibody used was a monoclonal mouse antibody raised against the N-terminal of purified CgA (M869; Dako, Carpinteria, CA, USA). The sections were incubated with primary antibody diluted to 1:1,000 at room temperature for 2 h. The sections were then washed in phosphate-buffered saline (PBS; pH 7.4) and incubated with biotinylated swine anti-mouse IgG (DakoCytomation, Glostrup, Denmark) diluted to 1:200 for 30 min at room temperature. Subsequent to washing the slides in PBS buffer, the sections were incubated for 30 min with avidin-biotin-peroxidase complex (DakoCytomation) diluted to 1:100 and then immersed in 3,3′-diaminobenzidine peroxidase substrate (Vector Laboratories), followed by counterstaining with haematoxylin.

### Computerised image analysis

The number of immunoreactive cells and the area of epithelial cells were measured using Olympus cellSens imaging software (version 1.7; Olympus, Tokyo, Japan) on a computer linked to an Olympus microscope (type BX 43) with built-in Koehler illumination for transmitted light, a light-intensity manager, a switchable high-colour-reproductivity LED light source, a 6 V/30 W halogen bulb and an Olympus camera (DP 26). The number of CgA-immunoreactive cells in each field was counted manually and the area of epithelium containing these cells was drawn manually using a computer. The intensity of CgA immunoreactivity in each field was measured using an automatic threshold setting. A ×40 objective was used, which resulted in each frame (field) on the monitor representing a tissue area of 0.035 mm^2^. For each subject, measurements were performed in ten randomly selected fields. Immunostained sections from the IBS patients and controls were coded and mixed, and measurements were conducted by the same individual who was blinded to the identity of the sections. The data from the fields were tabulated, and the cell density of the epithelium (in cells/mm^2^) and the immunoreactivity intensity were computed and analysed statistically.

### Statistical analysis

The differences in gender and the occurrence of *H. pylori* infection between patients and controls were examined using Fisher’s exact test, and the age difference was analysed by the Mann-Whitney nonparametric test. The differences among controls, all IBS patients (IBS-total), IBS-D, IBS-M and IBS-C patients were investigated using the Kruskal-Wallis nonparametric test with Dunn’s post test. The data are presented as the mean ± the standard error of the mean and P<0.05 was considered to indicate a statistically significant difference.

## Results

### Patients and controls

The gender and age distributions did not significantly differ between the patients and the controls (P=0.297 and P=0.824, respectively). *H. pylori* infection was identified in three of the patients and two of the control subjects (as evidenced by the urease test and by histopathological examination), and its incidence did not significantly differ between the two groups (P=0.64).

### Gastroscopy, histopathology and immunohistochemistry

The oesophagus, stomach and duodenum of the patients and the controls were macroscopically normal. Histopathological examination of the stomach revealed normal histology. CgA-immunoreactive cells were observed in the antra and corpora of patients and controls, and were basket- or flask-shaped, occasionally with a long basal cytoplasmic process.

### Computerised image analysis

**Figure 1:**
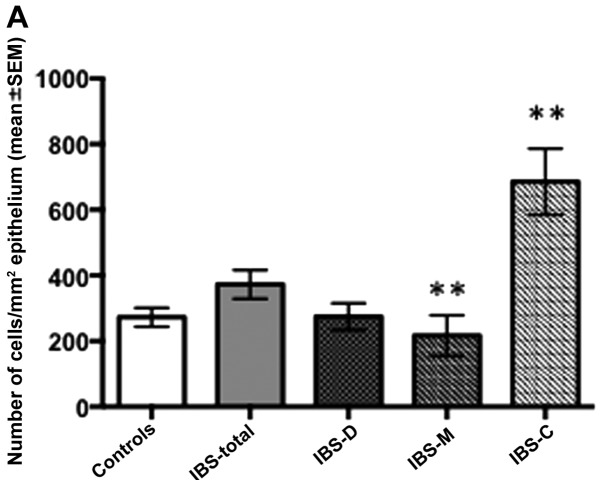

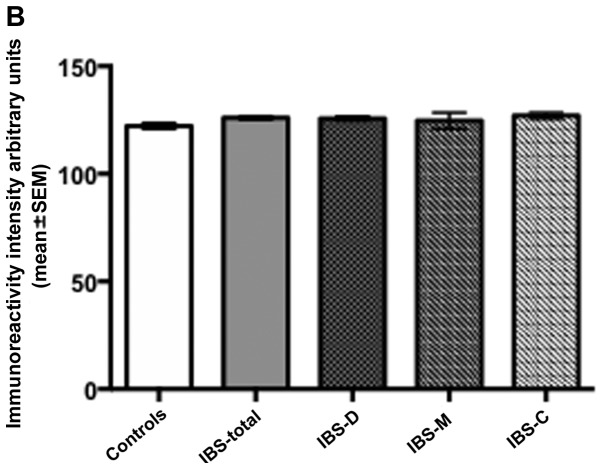
(A) CgA cell density and (B) intensity of anti-CgA immunoreactivity in the stomach antra of controls and of IBS-total, IBS-D, IBS-M and IBS-C patients. ^**^P<0.01 vs. controls. CgA, chromgranin A; IBS, irritable bowel syndrome; IBS-D, IBS diarrhoea; IBS-M, IBS diarrhoea and constipation; IBS-C, IBS constipation.

**Figure 2:**
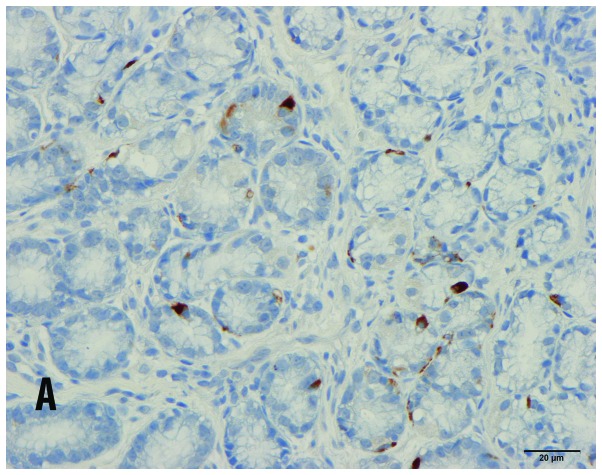

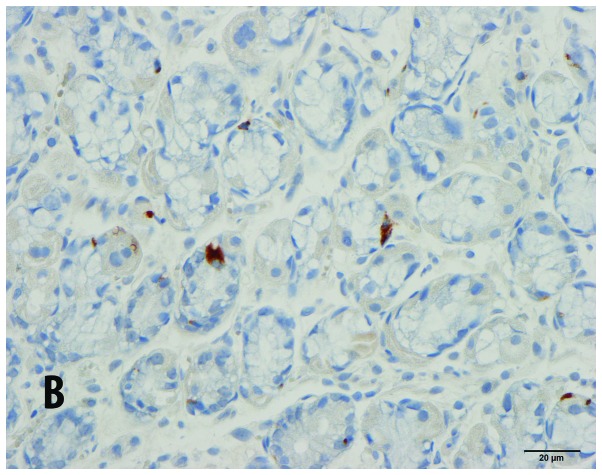

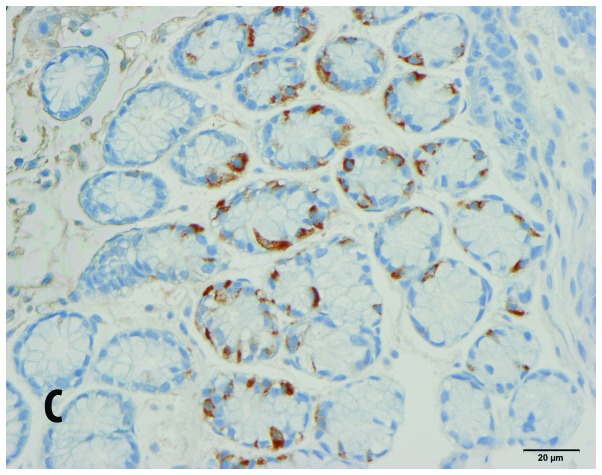
Antral chromogranin A-immunoreactive cells in (A) a control subject, (B) a patient with IBS-M and (C) a patient with IBS-C. Staining was performed using the avidin-biotin complex method. IBS, irritable bowel syndrome; IBS-M, IBS diarrhoea and constipation; IBS-C, IBS constipation.

In the antra, the densities of CgA cells were measured as 272.5±28.6, 372.7±43.6, 274.6±41.2, 217.0±61.9 and 685.3±100.5 cells/mm^2^ in the controls and the IBS-total, IBS-D, IBS-M and IBS-C patients, respectively. The Kruskal-Wallis test demonstrated that the CgA cell density differed significantly between the controls and the IBS-total and IBS subgroups (P=0.01). The Cga cell density was significantly lower in the IBS-M group and significantly higher in the IBS-C group relative to the controls (P=0.003 and P<0.01, respectively; Figs. 1 and 2). The intensities of the anti-CgA immunoreactivity were 122.2±1.4, 126.0±0.8, 125.7±0.9, 124.7±3.7 and 127.0±1.4, in the controls and the IBS-total, IBS-D, IBS-M and IBS-C patients, respectively. No significant differences in this parameter were detected among any of the groups (P=0.14; Figs. 1 and 2).

**Figure 3:**
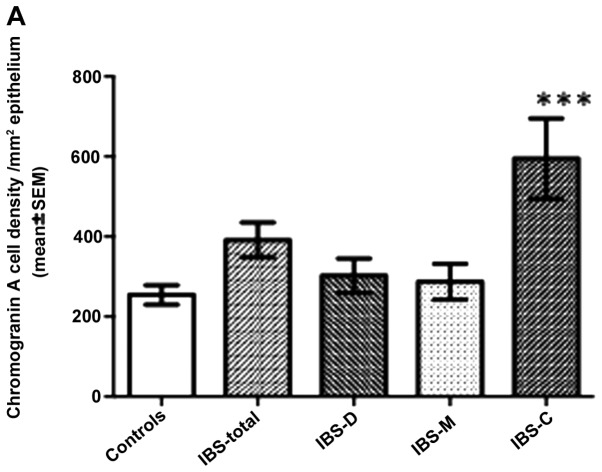

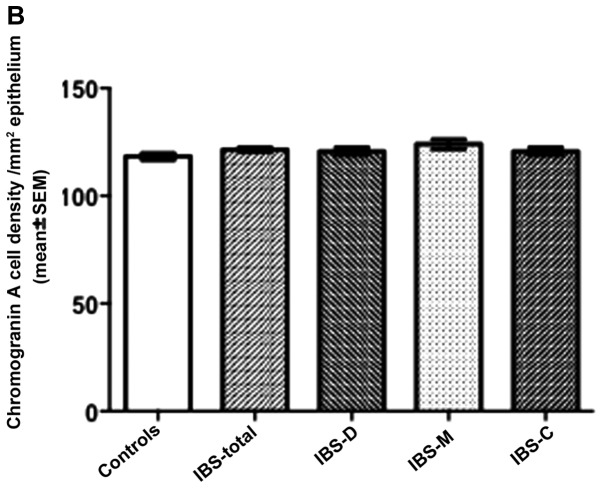
(A) CgA cell density and (B) intensity of anti-CgA immunoreactivity in the corpora of controls and of IBS-total, IBS-D, IBS-M and IBS-D patients. ^***^P<0.001 vs. controls. CgA, chromgranin A; IBS, irritable bowel syndrome; IBS-D, IBS diarrhoea; IBS-M, IBS diarrhoea and constipation; IBS-C, IBS constipation.

**Figure 4:**
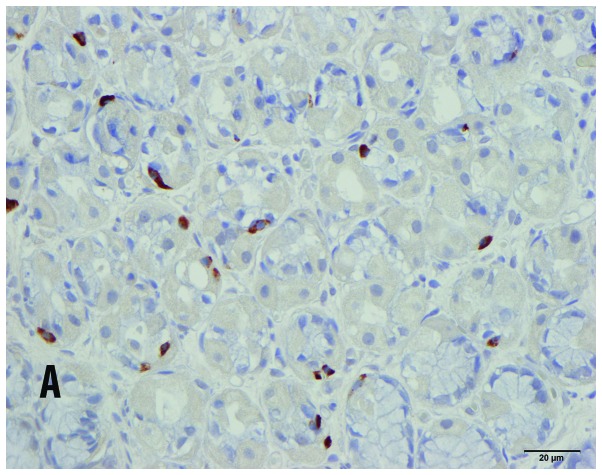

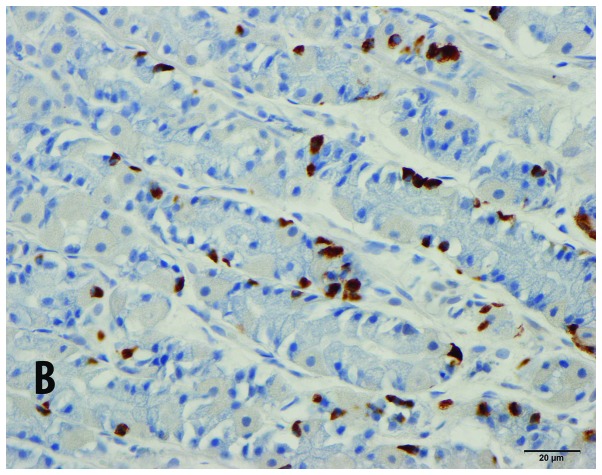
Chromogranin A cells in the corpora of (A) a control subject and (B) a patient with irritable bowel syndrome-C. Staining was performed using the avidin-biotin complex method.

In the corpora, the densities of CgA cells were 254.1±24.6, 391.3±43.4, 302.4±42.6, 286.9±44.9 and 594.7±100.1 cells/mm^2^ in the controls and the IBS-total, IBS-D, IBS-M and IBS-C patients, respectively. Multi-comparison with the Kruskal-Wallis test demonstrated that the CgA cell density differed significantly between the controls and the IBS-total and all IBS subgroups (P=0.004), and particularly between patients with IBS-C and the controls (P=0.0002; Figs. 3 and 4). The intensities of the CgA immunoreactivity were 118.3±1.6, 121.4±1.0, 120.7±1.7, 124.0±2.2 and 120.7±1.7 in the controls and the IBS-total, IBS-D, IBS-M and IBS-C patients, respectively. As in the antrum, this parameter did not differ significantly among any of the groups (P=0.29; Figs. 3 and 4).

## Discussion

The findings of the present study revealed that the density of all endocrine cells in the antrum and corpus of the stomach, as identified by CgA immunoreactivity, is abnormal in certain patients with IBS. This abnormality was not present for all IBS subtypes, the density of CgA-immunoreactive cells was increased in the antra and corpora of patients with IBS-C, and reduced in the antra of patients with IBS-M relative to the controls. Whereas CgA-immunoreactive cells in the antrum represent the population of gastrin-, serotonin- and somatostatin-secreting cells, in the corpus CgA-immunoreactive cells represent ghrelin-, serotonin- and somatostatin-secreting cells (1,15,16). Thus, although CgA cell density in the antrum and the corpus was unchanged in IBS-D patients, changes in particular endocrine cells cannot be excluded.

Computerised image analysis has been used for the past 15 years for determining the cellular concentrations of certain substances in a section of tissue by measuring the intensity of immunoreactivity produced by the colour of the immunohistochemical staining (17). When this practise was initially implemented, measuring these parameters was tedious, with instability in the light source also rendering the measurements unreliable. Subsequent advances in computer software and microscopic illumination have greatly improved the measurement reliability. However, the intensity of immunoreactivity is expressed in arbitrary units and thus is only useful for comparisons among groups immunostained under the same conditions. In the present study, no significant differences were detected in the intensities of CgA immunoreactivity in the antra and the corpora of the controls and IBS patients, including all IBS subtypes. This indicates that the cellular content of CgA was similar in the controls and all types of IBS patients. The cellular content of CgA reflects the synthesis and release of CgA, and the findings suggest that these functions are not affected in IBS patients.

With the exception of ghrelin in the oxyntic mucosa of the stomach of IBS patients (15,18), to the best of our knowledge the endocrine cells in the stomach of IBS patients have not been previously investigated. The density of ghrelin-secreting cells in the stomach was reported to be lower in IBS-C and higher in IBS-D than in healthy controls (19). Abnormalities in the endocrine cells in other parts of the gastrointestinal tract, including the duodenum, ileum, colon and rectum, have been reported in patients with IBS (12,13,19–30). In the duodenum, the cell densities of gastric inhibitory polypeptid- and somatostatin-secreting cells were reduced in IBS-D and IBS-C (21). The densities of duodenal secretin- and cholecystokinin (CCK)-secreting cells were reduced in IBS-D but unchanged in IBS-C, while the duodenal serotonin cells were not affected in either IBS-D or IBS (21). Furthermore, in postinfectious IBS, an increased number of duodenal CCK cells but a reduced number of serotonin-secreting cells have been reported (20). Densities of colonic serotonin- and peptide YY (PYY)-secreting cells were reportedly low in IBS-D and IBS-C (22). In the rectum, the densities of PYY and enteroglucagon-secreting cells were significantly lower and the density of somatostatin-secreting cells was significantly higher in IBS-D and IBS-C than in controls, whereas the density of serotonin-secreting cells in these patients did not differ from that in healthy controls (24,23). Densities of rectal serotonin- and PYY-secreting cells in post-infectious IBS were reported to be elevated (26,28,30,31,32).

In conclusion, the total numbers of endocrine cells were abnormal in the stomachs of patients with IBS. This abnormality was not associated with a change in the intensity of the immunoreactivity, possibly indicating unchanged synthesis and/or release of the hormones. In addition, the changes in endocrine cells observed in the present study varied among the IBS subtypes, which may indicate a role of the endocrine cells in differential symptomologies. The findings of the present study add to the evidence supporting the presence of endocrine cell abnormalities throughout the gastrointestinal tract in IBS patients.
